# Microbiological Safety of Leafy Vegetables Produced at Houeyiho and Sèmè-Kpodji Vegetable Farms in Southern Benin: Risk Factors for *Campylobacter spp.*

**DOI:** 10.1155/2019/8942608

**Published:** 2019-12-14

**Authors:** Sylvain Daton Kougblénou, Alidéhou Jerrold Agbankpé, Justin Gbèssohélé Béhanzin, Tamègnon Victorien Dougnon, Alidah Victonie Aniambossou, Lamine Baba-Moussa, Honoré Sourou Bankolé

**Affiliations:** ^1^Laboratory of Food Microbiology, Ministry of Health, 01 P.O. Box 418, Cotonou, Benin; ^2^Research Unit in Applied Microbiology and Pharmacology of Natural Substances, Research Laboratory in Applied Biology, Polytechnic School of Abomey-Calavi, University of Abomey-Calavi, 01 P.O. Box 2009, Cotonou, Benin; ^3^Laboratory of Molecular Physiopathology and Toxicology, Faculty of Science and Technology, University of Abomey-Calavi, 01 P.O. Box 4521, Cotonou, Benin; ^4^Laboratory of Biology and Molecular Typing in Microbiology, Faculty of Science and Technology, University of Abomey-Calavi, 05 P.O. Box 1604, Cotonou, Benin

## Abstract

Foodborne infections, mainly those attributable to *Campylobacter*, are one of the most common causes of intestinal diseases, of bacterial origin in humans. Although the vehicle of transmission is not always identified, the most common vehicles are poultry, poultry products, and contaminated water. In Southern Benin, an excessive use of poultry manure as fertilizer in vegetable farms was noted. This survey aimed to determine the prevalence and concentration of *Campylobacter spp.*, especially *Campylobacter jejuni* and *Campylobacter coli*, in selected environmental samples (poultry manure, and irrigation water) and freshly harvested leafy vegetables in two (Houeyiho and Sèmè-Kpodji) vegetable farms in southern Benin. To achieve this objective, we analyzed 280 samples, including 224 samples of leafy vegetables (*Solanum macrocarpon* and *Lactuca sativa capita*), 28 samples of irrigation water, and 28 samples of poultry manure. The analysis of the samples taken was carried out according to the modified NF EN ISO 10272-1 standard. Of the 280 samples analyzed in this survey, 63 were positive for *Campylobacter* contamination. For leafy vegetable samples analyzed in this survey, the contamination rate was of 15.63%. 60.71% of poultry manure samples analyzed were contaminated with *Campylobacter spp.* and 39.29% of irrigation water samples were contaminated. The statistical analysis of these results showed that there is a correlation between the contamination of leafy vegetables, poultry manure, and irrigations (*p* < 0.01). *Campylobacter jejuni* (53.97%) was more involved in contaminations than *Campylobacter coli* (36.57%). This study has shown that there is a real risk of food poisoning by *Campylobacter jejuni* and *Campylobacter coli* among consumers of leafy vegetables in southern Benin. The origin of contamination of these leafy vegetables is poultry manure used as fertilizer in vegetable gardens and irrigation water used.

## 1. Introduction

In Sub-Saharan Africa, the products of urban agriculture are considered to be one response to the shortage of foodstuffs [1]. In addition to the contributions of urban agriculture to urban food security, nutrition, and local economies, farming also affects urban water management, sanitation, and health services [2]. In that context, urban production of vegetables is increasing rapidly but, in both Africa and Asia, faces many constraints, especially land pressure, access to water, and low soil fertility [3, 4]. In Benin, West Africa, the same problems have been identified in periurban and urban gardening areas, where irrigated vegetable production developed rapidly after 1990, coinciding with the drastic drop in fish resources in the AtlanticOcean and the rivers [5]. Themain leafy vegetables grown are *Lactuca sativa* and *Solanum macrocarpon* L.

The emergence of coastal soil poverty and land pressure is leading farmers to intensify production using inorganic and organic fertilizers and pesticides. This in order to satisfy the growing demand for vegetables. Today, animal manure (60% poultry manure and 40% cattle manure) is frequently used as fertilizer in Southern Benin. Animal manures have been used as effective fertilizers for centuries [5, 6]. Brooks et al. [7] investigated potential microbial runoff associated with the application of poultry litter on the soil. Several other studies pointed to pollution and health risks caused by lack of knowledge and bad practices in the management of livestock manure and chemical fertilizers [1, 3, 8].

Excessive use of fertilizer at each agricultural campaign has been reported in both Africa and Asia, particularly the use of poultry manure at rates of 20-50 t·ha^−1^ and the use of mineral fertilizers, such as urea and NPK (10-20-20 nitrogen, phosphorus, and potassium fertilizer), at rates of 1.2-2 t·ha^−1^ [8, 9]. Unfortunately, the intensive use of organic matter like cow dung and poultry manure and other animal feces are a significant environmental risk to soils, waters, and crops, including fecal contamination [8].

Researchers at Emerging Pathogens Institute (EPI) at the University of Florida in the United States recently focused on Infectious Diseases of Food Origin. They estimated that 31 food-borne pathogens are responsible for 9.4 million human infections each year in the United States, resulting in 55.961 hospitalizations and 1.351 deaths. Of all these cases, 39% are associated with bacteria, including *Campylobacter*, *Salmonella*, and *Clostridium perfringens*, which occupy the top three positions in the ranking [10].

Campylobacteriosis is a serious global issue owing to the heavy economic burden caused by the disease. In the United States, as much as US$ 4.3 billion is estimated to be used in fighting against this disease in one year [11]. The epidemiology of *Campylobacter* infections in humans is not well understood, yet campylobacteriosis is known to be sporadic and rarely associated with large outbreaks. Although the vehicle of transmission is not always identified [12], the most common vehicles are poultry, poultry products, raw milk, and contaminated drinking water [12, 13]. Produce as a potential route for transmitting foodborne diseases to humans has recently gained more attention owing to changes in diet [14]. As the overall consumption of fresh produce, especially raw vegetables, has increased as a result of an increase in health consciousness, raw vegetables could serve as a potential vehicle for the transmission of foodborne pathogens to humans. Although *Campylobacter spp.* are not usually detected in produce or other produce-related products [15, 16], the Centers for Disease Control and Prevention [17] has a record of 18 outbreaks of *Campylobacter enteritis* associated with produce worldwide from 1990 to 1999, and the first reported *Campylobacter* outbreak associated with fresh produce occurred in 1993 in the United States and was linked to melons and strawberries [17].

Accordingly, the present study was undertaken to evaluate the level of contamination of leafy vegetables produced in southern Benin by *Campylobacter jejuni* and *Campylobacter coli*.

## 2. Methodology

### 2.1. Choice of Sampling Sites

The samples were taken come from vegetable farms of the communes of Sèmè-Kpodji and Cotonou. These are the two largest vegetable sites in southern Benin with an area of about 80 hectares for the Sèmè-Kpodji vegetable site [18] and more than 15 hectares for the Houéyiho [19] in Cotonou.

### 2.2. Sampling

#### 2.2.1. Sample Size

The minimum size (n) of the samples was estimated from Schwartz formula: *n* = (*z*^2^ × *p* × *q*)/*d*^2^ where *n* is the minimum size of the samples; *p* (prevalence) = 0.20 because the prevalence of *Campylobacter spp.* in Benin is 20% in poultry meat samples [20]; *q* = 1 − *p* = probability that a sample is not contaminated with *Campylobacter spp.*; *z* = confidence level according to the normal reduced centered law (for a 95% confidence level, *z* = 1.96, *d* = a margin of error tolerated for this survey equal to 0.05, the minimum size of the samples is therefore: *n* = (1, 96^2^ × 0.2 × 0.8)/0, 05^2^ = 245, 86. Let *n* = 256 samples.

The total number of samples selected for this study is *N* = 280. These samples consist of leafy vegetables (*Solanum macrocarpon* L. and *Lactuca sativacapitata*), irrigation water and poultry manure. *Solanum macrocarpon* was chosen because it is the most widely grown leaf vegetable on both sampling sites. The choice of *Lactuca sativa capitata* was motivated by its culinary technique.

#### 2.2.2. Sampling Technique

Market gardens where poultry manure is used have been identified. At random, 30 gardens were selected on each of Sèmè-Kpodji and Houeyiho vegetable farms, at a rate of one garden per hectare and at a distance of at least 20 m from each other. On Sèmè-Kpodji vegetable farm, 8 gardens using poultry manure and 8 using NPK (10–20–20 nitrogen, phosphorus, and potassium fertilizer) as fertilizer were thus selected to undergo the sampling. On the other hand, on Houeyiho vegetable farm, for each type of garden, 6 gardens were selected.

By garden using poultry manure as fertilizers, 4 samples of *Solanum macrocarpon*, 4 samples of *Lactuca sativa capitata*, 2 poultry manure samples and 1 irrigation water samples were taken. The same was true for gardens using only NPK as fertilizer (Table 1). The leafy vegetables were cut with a pair of sterile scissors and forceps about 5 cm from the root. A mass of about 300 g of fresh leaves was thus taken from the vegetable plants and introduced into two sterile plastic bags.

Using a sterile spoon, about 300 g of poultry manure was collected in sterile plastic bags. Irrigation water samples were collected by immersing one-liter sterile glass bottles in water.

All samples thus taken were numbered and immediately sent to a laboratory in a cooler containing cold accumulators for analysis. Microbiological analyses were performed within 4 hours after sampling.

#### 2.2.3. Enrichment, Isolation, and Purification of Campylobacter spp. Strains

The analysis of the samples taken were carried out according to the modified NF EN ISO 10272-1 standard described by Bankolé et al. [20]. 25 g of sample was taken in a sterile bag containing 225 mL Preston broth (Oxoid, England) enriched with fresh sheep blood and Preston supplement (Oxoid CM0689, England). After homogenization with Stomacher, the bag was then hermetically closed.

Assembly obtained was incubated at 42°C ± 1°C under microaerophilic condition (incubation in a jar containing a lit candle) for 48 hours ± 2 h. Subsequently, 48 h ± 2 h subculture was streak-seeded on Preston-Campylobacter (PC) and Karmali-Campylobacter (KC) agar plates. These plates were incubated microaerophilic condition at 42°C ± 1°C for 48 h ± 2 h. After incubation, a characteristic *Campylobacter* colony was taken from PC and KC agars respectively and seeded on nutrient agar (NG) enriched with fresh sheep blood. These agar plates then were incubated microaerophilic condition at 37°C for 36 h ± 2 h. The pure cultures obtained were stored in glycerol MH broth (30%) at −37°C (for two weeks) for further analyses.

#### 2.2.4. Phenotypic Identification of Campylobacter spp. Strains

Identification of *Campylobacter spp.* strains was carried out based on bacterial strain morphology, Gram stain, biochemical characterization tests (catalase, oxidase, hydrolysis of hippurate, nitrate production reductase, fermentation of sugars, production of hydrogen sulphide and gas and growth at 25°C and 42°C and antibiotypage). These biochemical tests was carried out according to NMKL 119 [21]. *Campylobacter spp.* isolates were identified as *C. jejuni, C. coli *or *Campylobacter spp.* Reference strains of *Campylobacter* (*Campylobacter jejuni* ATCC 29428, *Campylobacter coli* ATCC 33559) and other bacteria (*Pseudomonas aeruginosa* ATCC 27853, *Staphylococcus aureus* ATCC 29213, *Escherichia coli *ATCC 25922) were used to validate the tests and techniques used.

## 3. Results

### 3.1. Samples Contamination of Sèmè-Kpodji Vegetable Farm

The samples of the garden using poultry manure as fertilizer had a contamination rate of 27.3%. Of the 64 leafy vegetable samples, 12 (18.8%) were *Campylobacter spp.* positive, and of the 16 poultry manure samples, 9 (56.3%) were *Campylobacter spp.* The bivariate correlation analysis between the contamination rate of poultry manure and leafy vegetables showed a significant correlation at the 0.01 level (*p*-value ≤ 0.001). Samples from gardens using NPK fertilizer had a contamination rate of 11.1%. The leafy vegetable and irrigation water samples had a contamination rate of 7.8% and 37.5%, respectively. There is a significant correlation at 0.01 level between the contamination of irrigation water and that of leafy vegetables (*p*-value ≤ 0.001) (Table 2).

### 3.2. Contamination of Samples from Houeyiho (Cotonou) Vegetable Farm

25.8% (31/120) of samples taken from Houeyiho vegetable farm were contaminated with *Campylobacter spp.* Contamination rates of samples from gardens using poultry manure and NPK as fertilizer were, respectively, 33.3% and 16.7%. On gardens using poultry manure, 25% (12/48) and 66.7% (8/12) of leafy vegetables and poultry manure were positive for *Campylobacter spp.* Analysis of these results showed a significant correlation at 0.01 level between contaminated poultry manure and contaminated leafy vegetables (*p-*value ≤ 0.001). For gardens using NPK, contamination rate of leafy vegetable and irrigation water samples were respectively 12.5% (6/48) and 50% (3/6). There is also a significant correlation at 0.01 level between contamination rates of irrigation water and leafy vegetable samples (*p*-value ≤ 0.001) (Table 3).

With regard to contamination of two leafy vegetable species contaminated by *Campylobacter spp.*, the contamination rates of 10.7% (12/112) and 20.5% (23/112) were recorded for *Solanum macrocarpon* and *Lactuca sativa* capita, respectively. This contamination of these leafy vegetable species does not depend on the type of garden nor vegetable farms (Table 4).

### 3.3. Distribution of Campylobacter Species to Contaminated Samples from Sèmè-Kpodji Vegetable Farm

Of the 32 contaminated samples from Sèmè-Kpodji vegetable farm, 53.1% were contaminated with *Campylobacter jejuni*, 37.5% with *Campylobacter coli* and 9.4% with other *Campylobacter* species.

**Figure 1:**
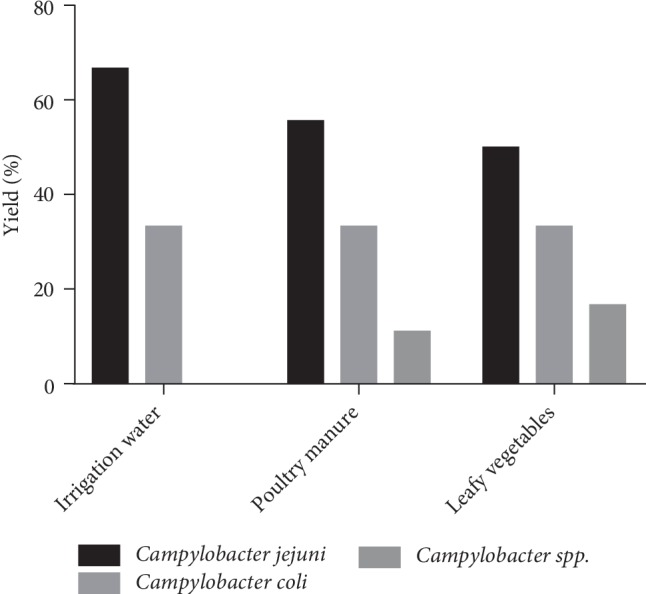
Distribution of *Campylobacter* species from contaminated garden samples using poultry manure.

Regarding the numbers of contaminated samples from gardens using poultry manure as fertilizer, we noted that 50% of leafy vegetable samples were contaminated by *Campylobacter jejuni*, 33.3% by *Campylobacter coli* and 16.7% by *Campylobacter spp.* Of the poultry manure, 55.6% were contaminated with *Campylobacter jejuni*, 33.3% with *Campylobacter coli* and 11.1% with *Campylobacter spp.* For the irrigation water samples, 66.7% were contaminated with *Campylobacter jejuni* and 33.3% with *Campylobacter coli* (Figure 1). Bivariate correlation analysis showed a significant correlation at 0.01 level of *Campylobacter* species distribution between leafy vegetable and poultry manure samples contaminated (*PC* = 0.875, *p*-value = 0.002).

**Figure 2:**
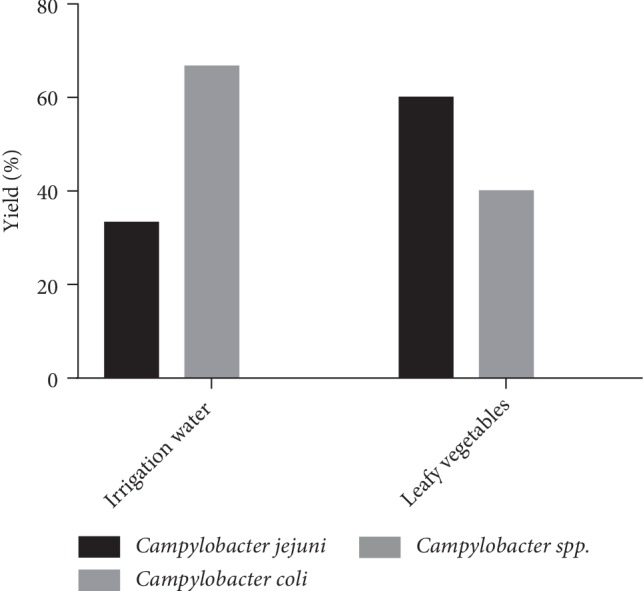
Distribution of *Campylobacter* species by to NPK Gardens contaminated samples.

In gardens where NPK is used as a fertilizer, *Campylobacter jejuni* was present in 60% of leafy vegetable samples contaminated and 40% *Campylobacter coli* contamination. For contaminated irrigation water samples, there is a high proportion of *Campylobacter coli* (66.7%) and a low proportion of *Campylobacter jejuni* (33.3%). The samples from these gardens are only contaminated by these two species of *Campylobacter *(Figure 2). But there is no significant correlation between these samples at 0.01 level (*PC* = 0.500, *p-*value = 0.667).

### 3.4. Distribution of Campylobacter Species according to Contaminated Samples from Houehiyo (Cotonou) Vegetable Farm

**Figure 3:**
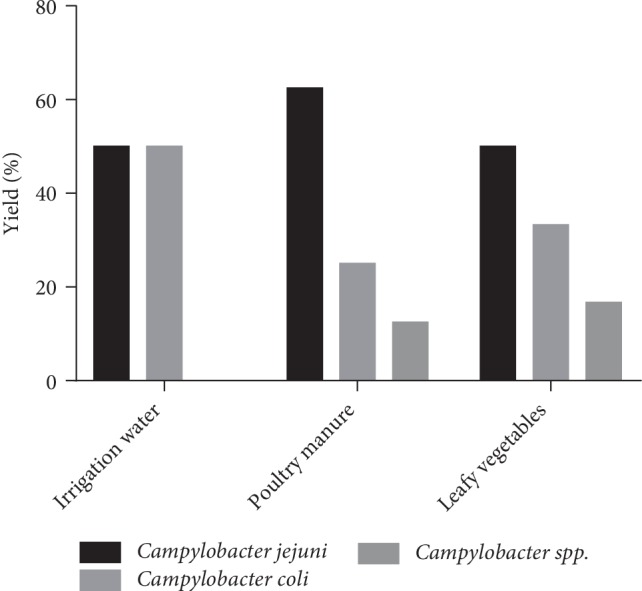
Distribution of *Campylobacter* species by contaminated samples from gardens using poultry manure.

Seventeen contaminated samples from Houehiyo vegetable farm were by *Campylobacter jejuni* (54.8%). *Campylobacter coli* contamination accounted for 35.5% and that for other unidentified *Campylobacter* species accounted for 9.68%. In gardens using poultry manure as fertilizer, contaminated leafy vegetable samples were 50% *Campylobacter jejuni*, followed by 33.3% *Campylobacter coli* and *Campylobacter spp.* at 16.7%. 62.5% of the contaminated poultry manure were *Campylobacter jejuni*, 25% by *Campylobacter coli* and 12.5% by *Campylobacter spp.* Contaminated irrigation water samples were 50% *Campylobacter jejuni* and *Campylobacter coli* (Figure 3). There is a significant correlation at 0.01 level of distribution of *Campylobacter* species between contaminated leafy vegetable and poultry manure samples (*PC* = 1; *p*-value ≤ 0.001).

**Figure 4:**
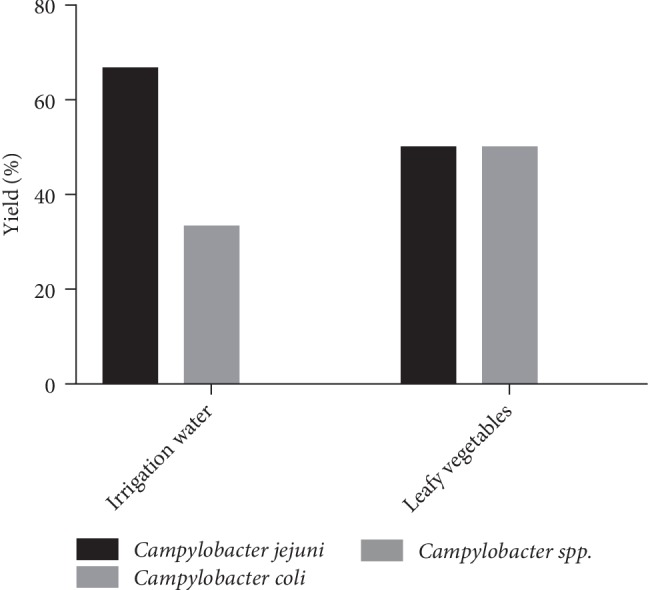
Distribution of *Campylobacter* species according to contaminated samples from NPK gardens.

With regard to contaminated samples from gardens using NPK fertilizer, *Campylobacter jejuni*, and *Campylobacter coli* are the only species present. The contaminated leafy vegetable samples were 50% *Campylobacter jejuni* and *Campylobacter coli*. As for the irrigation water samples, 66.7% were contaminated with *Campylobacter jejuni* and 33.3% with *Campylobacter coli* (Figure 4). The statistical analysis of these results showed that there is a significant correlation at 0.01 level between leafy vegetable contamination by *Campylobacter jejuni*, *Campylobacter coli* and that of irrigation water (*PC* = 1, *p*-value ≤ 0.001).

## 4. Discussion

The contamination rate of 22.5% obtained in this study was a relatively high contamination rate; nevertheless, it indicates the presence of *Campylobacter* on these two vegetables farms. This rate of *Campylobacter* contamination is somewhat similar to that of imported poultry meat (20%) in Benin [20].

The contamination rate of vegetables samples analyzed (15.6%) seems high to that obtained by Chai et al. [23] on Malaysian farms where 6.3% of vegetables were contaminated with *Campylobacter spp.* This contamination rate, nevertheless confirms Jacobs-Reitsma hypothesis, which at the end of its work in 2000 reached the same conclusion that leafy vegetables could be contaminated by *Campylobacter spp.* [24]. Comparison of results of contaminated leafy vegetables shows that leafy vegetables from gardens amended with poultry manure (21.4%) are twice as contaminated with *Campylobacter* as those from gardens using NPK (9.8%) as fertilizers. This difference in the level of contamination could be explained by the nature of the fertilizers used.

The results about poultry manure contaminated (60.7%) confirm the observations made by several authors at the end of their respective studies. This is the case of Shanker et al., who demonstrated during their work in 1990, the presence of *Campylobacter* in 53.5% of the 174 poultry farms visited and who also showed during their study that, of these contaminated farms, 43.3% were found in more than half of the collected droppings [25]. This is also what emerges from the work of Yan et al. [26]. According to Atidegla et al. [5], contamination of leafy vegetables by pathogenic bacteria at vegetable farms in Benin is mainly due to the use of poultry manure. This observation is the same at both farms where our survey was conducted. In addition, a significant correlation at 0.01 level was noted between contaminated leafy vegetables and poultry manures contaminated with *Campylobacter spp.* (*PC* = 0.878 and 1.000, *p*-value ≤ 0.001). From these results, it follows that poultry manure is a likely source of contamination of leafy vegetables by *Campylobacter spp*.

However, would poultry manure be the only source of contamination of leafy vegetables? Leafy vegetable samples from gardens amended with NPK are contaminated. Similarly, irrigation water used to water leafy vegetables in these gardens are contaminated (42.9%). In addition, they are acorrelation of leafy vegetables and irrigation water samples from gardens using NPK fertilizer (*PC* = 1.000, *p*-value ≤ 0.001). So, it can be said, like poultry manure, that irrigation water would also be a potential source of contamination by *Campylobacter spp.* This is especially true since most of the irrigation water used in all these gardens is marigot or wells water about 1 - 2 m deep, and therefore exposed to all kinds of pollution including *Campylobacter* contamination. This observation is similar to the results of a study conducted in 2001 by Savill et al. in New Zealand in groundwater [27]. Our results are in line with those obtained in 2002 by Schaffer and Parriaux who, at the end of their studies, revealed the presence of *Campylobacter spp.* in surface water and runoff [28]. The same observation was made by Lyngstard et al. [29] and Sparks [30] who also showed that poor quality water (untreated water from wells) may contain *Campylobacter spp.* Other authors, including Hanninen et al. have shown that water can also be an epidemic source of campylobacteriosis, particularly in countries where there is insufficient chlorination of drinking water [31]. In addition, several authors have shown through the results of their work that pigs are essential reservoirs of *Campylobacter*. This is the case of the work done by Weijtens et al. (2000), which showed on 10 pigs in 8 farms, that 85–90% of these animals, according to age, are positive with a level of excretion of *Campylobacter* by the sows which increases after the farrowing [32]. While in some vegetable farms, including Houeyiho site, some market gardeners practice pig farming, in addition, more seriously, some hen pens are near irrigation points. This would certainly be the cause of the high levels of contamination (50%) obtained in gardens where NPK is used as fertilizer.

The difference of contamination rate between the two leafy vegetable types (*Solanum macrocarpon* and *Lactuca sativa capitata*) could be explained by the particular architecture of *Lactuca sativa capitata*. A provision that gives them a high capacity for water retention, unlike *Solanum macrocarpon*. This arrangement also increases the water content of the lettuce and thus promotes the survival of *Campylobacter spp.* for several days. This hypothesis is supported by the work of several researchers. This is the case of Daczkowska-Kazon and Brzostek-Nowakowska who have shown that some *Campylobacter* species including *Campylobacter jejuni* and *Campylobacter lari* appear to be more resistant in river water [33] and Talibart et al. (2000) who revealed that survival times of *Campylobacter spp.* in water could be very variable depending on the strains (from 6 to more than 60 days) [34]. In addition, Trigui et al. (2015) have shown that *Campylobacter spp.* can survive and remain viable in water for long periods of time (30 to 52 days) [35]. Often consumed in the “raw state” consumers of *Lactuca sativa capitata*, are truly more exposed to a *Campylobacter *infections than those who consume *Solanum macrocarpon*.

The statistical analysis of results showed that there is a significant correlation at 0.01 level of distribution of two *Campylobacter* species between leafy vegetable and poultry manure samples (*PC* = 0.875 and 1.000; *p*-value ≤ 0.001 and *p*-value = 0.002) and between leafy vegetable and irrigation water samples (*PC* = 1.000, *p*-value ≤ 0.001). There is strong contamination of leafy vegetables by *Campylobacter jejuni* than *Campylobacter coli* shares. But the statistical analysis of our data did not show a significant difference. Mohammadpour et al. (2018) [36] obtained results different from ours, where 18.2% and 2.5% fresh vegetables were contaminated, respectively, by *Campylobacter jejuni* and *Campylobacter coli*.

## 5. Conclusion

The present work showed on all samples analyzed a contamination rate by *Campylobacter spp.* of 22.5%. The leafy vegetable samples analyzed were contaminated with *Campylobacter spp.* at a rate of 15.6%. This contamination rate is apparently low, but shows that there is a risk of food poisoning by *Campylobacter spp.* in consumers of these leafy vegetables. Even so, these leafy vegetables are often eaten raw. With regard to the origin of this contamination, poultry manure used as fertilizer and the irrigation water used in the gardens of the selected vegetable farms are incriminated. *Campylobacter jejuni* was much more identified (51.4%) as a species involved in the contamination of leafy vegetables, followed by *Campylobacter coli* (37.1%). However, this difference is not significant.

## Figures and Tables

**Table 1:** Distribution of samples from Sèmè-Kpodji and Houeyiho (Cotonou) vegetable farms.

Farms	Type of garden	Nature of samples		Effective
Sèmè-Kpodji	Manure-garden	Leafy vegetables	*Solanum macrocarpon*	32
			*Lactuca sativa capitata*	32
		Irrigation water		08
		Poultry manure		16
	NPK-garden	Leafy vegetables	*Solanum macrocarpon*	32
			*Lactuca sativa capitata*	32
		Irrigation water		08
Effective Sèmè-Kpodji samples				160

Houeyiho	Manure-garden	Leafy vegetables	*Solanum macrocarpon*	24
			*Lactuca sativa capitata*	24
		Irrigation water		06
		Poultry manure		12
	NPK-garden	Leafy vegetables	*Solanum macrocarpon*	24
			*Lactuca sativa capitata*	24
		Irrigation water		06
Effective Houeyiho samples				120

Total number of samples				280

Manure-gardens: gardens where poultry manure are used as fertilizer; NPK-gardens: Gardens where NPK are used as fertilizer.

**Table 2:** Contamination rate of samples from Sèmè-Kpodji vegetable farm.

	Samples	Analysis results		Number of samples	*PC*	*p*-value
		+	−			
Manure-garden	Leafy vegetables	12 (18.8%)	52 (81.3%)	64 (72.7%)	0.878	0.001
	Poultry manure	9 (56.3%)	7 (43.8%)	16 (18.2%)		
	Irrigation water	3 (37.5%)	5 (62.5%)	8 (9.1%)		
	Total number of samples	24 (27.3%)	64 (72.7%)	88 (100%)		

NPK-garden	Leafy vegetables	5 (7.8%)	59 (92.2%)	64 (88.9%)	1.000	0.001
	Irrigation water	3 (37.5%)	5 (62.5%)	8 (11.1%)		
	Total number of samples	8 (11.1%)	64 (88.9%)	72 (100%)		

Manure-garden: gardens where poultry manure are used as fertilizer; NPK-garden: gardens where NPK are used as fertilizer; +: positive; −: negative; *PC*: *Pearson correlation*.

**Table 3:** Contamination rate of samples from Houeyiho vegetable farm.

	Samples	Analysis results		Number of samples	*PC*	*p*-value
		+	−			
Manure-garden	Leafy vegetables	12 (25%)	36 (75%)	48 (72.7%)	1.000	0.001
	Poultry manure	8 (66.7%)	4 (33.3%)	12 (18.2%)		
	Irrigation water	2 (33.3%)	4 (66.7%)	6 (9.1%)		
	Total number of samples	22 (33.3%)	44 (66.7%)	66 (100%)		

NPK-garden	Leafy vegetables	6 (12.5%)	42 (87.5%)	48 (88.9%)	1.000	0.001
	Irrigation water	3 (50%)	3 (50%)	6 (11.1%)		
	Total number of samples	9 (16.7%)	45 (83.3%)	54 (100%)		

Manure-garden: gardens where poultry manure are used as fertilizer; NPK-garden: gardens where NPK are used as fertilizer; +: positive; −: negative; *PC*: *Pearson correlation*.

**Table 4:** Contamination rate of two leafy vegetable species sampled.

Vegetable farms	Type of garden	Number of samples contaminated by *Campylobacter spp.* according to each leafy vegetables				Contamination rate of leafy vegetables by type of gardens	Contamination rate of leafy vegetables by farms	Total
		*Solanum macrocarpon*		*Lactuca sativa capitata*				
Sèmè-Kpodji	Manure-gardens	3	(25%)	9	(75%)	12 (18.75%)	17 (13.28%)	**128**
	NPK-gardens	2	(40%)	3	(60%)	5 (7.81%)		
Houeyiho	Manure-garden	4	(33.33%)	8	(66.67%)	12 (25%)	18 (18.75%)	**96**
	NPK-garden	3	(50%)	3	(50%)	6 (12.50%)		
Total		**12/112**	**(10.71%)**	**23/112**	**(20.54%)**			

## Data Availability

The data used to support the findings of this study are included within the article.
